# Expression of AE1/p16 promoted degradation of AE2 in gastric cancer cells

**DOI:** 10.1186/s12885-016-2751-x

**Published:** 2016-09-05

**Authors:** Ting Wang, Hong-Jun Fei, Ye Yang, Xiao-Shu Jiang, Min Yan, Zhi Zeng, Jun Wu, Ling-Jun Song, Hua Tian, Guo-Hui Fu

**Affiliations:** 1 Pathology Center, Shanghai General Hospital/Faculty of Basic Medicine, Shanghai Jiao Tong University School of Medicine, No.280, South Chong-Qing Road, Shanghai, 200025 People’s Republic of China; 2 Department of Digestive Medicine, Ningbo No. 2 Hospital, Ningbo, 315010 People’s Republic of China; 3 Department of Pathophysiology, Harbin Medical University, Harbin, 150081 People’s Republic of China; 4 Department of General Surgery, Ruijin Hospital, Shanghai Jiao Tong University School of Medicine, Shanghai, 200025 People’s Republic of China; 5 State Key Laboratory of Oncogenes and Related Genes, Shanghai Cancer Institute, Renji Hospital, Shanghai Jiao Tong University School of Medicine, Shanghai, 200032 People’s Republic of China

**Keywords:** AE1, AE2, p16, Gastric cancer, Gastrin

## Abstract

**Background:**

Human anion exchanger 1 and 2 (AE1 and AE2) mediate the exchange of Cl^−^/HCO_3_^−^ across the plasma membrane and regulate intracellular pH (pHi). AE1 is specifically expressed on the surface of erythrocytes, while AE2 is widely expressed in most tissues, and is particularly abundant in parietal cells. Previous studies showed that an interaction between AE1 and p16 is a key event in gastric cancer (GC) progression, but the importance of AE2 in GC is unclear.

**Methods:**

The relationship among AE1, AE2 and p16 in GC cells was characterized by molecular and cellular experiments. AE2 expression and pHi were measured after knockdown or forced expression of AE1 or p16 in GC cells. The effect of AE2 on GC growth and the correlation of AE2 expression with differentiation and prognosis of GC were also evaluated. The effect of gastrin on AE2 expression and GC growth was investigated in cellular experiments and mouse xenograft models.

**Results:**

p16 binds to both AE1 and AE2 simultaneously. AE1 or p16 silencing elevated AE2 expression on the plasma membrane where it plays a role in pHi regulation and GC suppression. AE2 expression was decreased in GC tissue, and these decreased levels were correlated with poor differentiation and prognosis of GC. The low AE2 protein levels are due to rapid ubiquitin-mediated degradation that was facilitated in the presence of p16. Gastrin inhibited the growth of GC cells at least partially through up-regulation of AE2 expression.

**Conclusion:**

AE1/p16 expression promoted AE2 degradation in GC cells. Gastrin is a potential candidate drug for targeted therapies for AE1- and p16-positive GC.

## Background

Gastric cancer (GC) is the fourth most common cancer and the third most common cause of cancer-related deaths worldwide. Despite advances in GC prevention and treatment, the 5-year survival rate for this cancer remains at 20–25 % [1]. GC can be classified into intestinal (well- or moderately-differentiated) and diffuse (poorly-differentiated) types based on histopathological characteristics [2, 3]. In general, intestinal GC is associated with gastritis that might progressively lead to atrophy and metaplasia, dysplasia and finally cancer. Although gastritis may, in some cases, account for diffuse types of GC, this GC type does not arise via the above-described cascade of histological events [4–6].

The AE family consists of AE1, AE2 and AE3 polypeptides that mediate electroneutral, Na^+^-independent, plasmalemmal Cl^−^/HCO_3_^−^ exchange [7]. AE1 (also known as band 3 or SLC4A1) is specifically expressed on the surface of erythrocytes, where it constitutes nearly 50 % of the total integral membrane proteins [8, 9]. Meanwhile, AE2 (SLC4A2) regulates intracellular pH (pHi), intracellular chloride concentration, bicarbonate metabolism and cell volume in a variety of cell types, but is most abundant in gastric parietal cells [10–12], where AE2 is required for the secretion of gastric acid to mediate basolateral Cl^−^ uptake, with additional contributions from the Cl^−^/HCO_3_^−^ exchanger SLC26A7 and Na/K/2Cl cotransporter, NKCC1 [13–16].

We previously found that AE1 is expressed in the cytoplasm of GC cells and its C-terminal 112 residues interacted with the tumor suppressor p16 [17, 18]. The cytoplasmic AE1/p16 complex enhanced the stability of both proteins and played a key role in GC progression, which was correlated with short survival time of GC patients [19–21]. In addition, siRNA-mediated suppression of AE1 significantly reduced the detection rate of GC in an *H. pylori*-induced animal model of GC [22]. In this study, we revealed that p16 binds not only AE1 but also AE2, and that the formation of the AE1/p16 complex accounted for the enhanced degradation of AE2 in poorly differentiated GC cells. Moreover, gastrin, a major gastrointestinal hormone, could inhibit GC growth by blocking AE1/p16-promoted AE2 degradation.

## Methods

### Cell culture and reagents

Human HEK293T cells, GC SGC7901 cells (AE1- and p16-positive) and MKN28 cells (AE1- and p16-negative) were cultured in DMEM/RPMI-1640 (Hyclone, Logan, UT, USA) containing 10 % fetal bovine serum (FBS, Hyclone) and 1 % penicillin/streptomycin (Invitrogen, Carlsbad, CA, USA) in an atmosphere of 5 % CO_2_ at 37 °C. The following reagents were purchased from the indicated companies: 2′,7′-bis-(2-carboxyethyl)-5-(and-6)-carboxyfluorescein acetoxymethyl ester (BCECF-AM) (Dojindo Laboratories, Kumamoto, Japan), Cycloheximide (CHX) (Sigma-Aldrich, St. Louis, MO, USA), MG132 (Merck KgaA Darmstadt, Germany) and 3-(4,5-dimethyl-2-thiazolyl)-2,5-diphenyl-2-H-tetrazolium bromide (MTT) (Sigma-Aldrich). Antibodies used for immunoblot analysis or IP experiments were included as follows: anti-p16 (Santa Cruz, CA, USA; BD Pharmingen, USA), anti-GFP (Santa Cruz), anti-HA (Santa Cruz) and anti-β-actin (Sigma-Aldrich). The antibody used for immunofluorescence and immunohistochemistry was anti-AE2 (Sigma-Aldrich).

### Co-immunoprecipitation (co-IP) and immunoblot analysis

SGC7901 cells were lysed with Radioimmunoprecipitation assay (RIPA) lysis buffer (Beyotime Institute of Biotechnology, Shanghai, China) containing fresh protease inhibitors and PMSF. The lysates were then incubated with anti-p16 antibody (BD Pharmingen) overnight at 4 °C, followed by incubation with Protein G Plus/Protein A Agarose Suspension (Merck) for another 4 h at 4 °C. After washing 3 times with the ice-cold lysis buffer, proteins were released from the beads using SDS lysis buffer for 10 min at 95 °C and then resolved on 10 % SDS-PAGE gels and analyzed by immunoblotting. To block the nitrocellulose membranes, 5 % skimmed milk in TBST was used to reduce nonspecific background. Membranes were then incubated with primary antibodies overnight at 4 °C. After washing in TBST 3 times for 10 min each, membranes were incubated with secondary antibodies for 1 h at room temperature, and then washed again as before. Bound antibodies were detected using a chemiluminescence phototope-horseradish peroxidase kit according to the manufacturer’s instructions (Pierce, Rockford, IL, USA).

### Ubiquitination assay

SGC7901 cells were transfected with pEGFP-AE2a, HA-Ub and p16 expression plasmids as indicated in Fig. 3c. After transfection for 42 h, cells were treated with 10 μM MG132 for an additional 6 h and then harvested and lysed in RIPA lysis buffer containing fresh protease inhibitors and PMSF. Cell extracts were incubated with anti-GFP antibody overnight at 4 °C, followed by incubation with the beads for another 4 h at 4 °C. After separation from the beads, the proteins were resolved by 8 % SDS-PAGE gels and analyzed by immunoblotting with anti-Ub antibody to detect ubiquitination.

### Cell fractionation

SGC7901 cells were transfected with AE1- or p16-targeted siRNA or shRNA plasmids separately. The cells were then harvested and the cell fractionation experiment was carried out using a Membrane and Cytosol Protein Extraction Kit (Beyotime Biotechnology, China) according to the manufacturer’s instructions.

### Immunofluorescence

SGC7901 cells were seeded onto glass coverslips and allowed to adhere for 24 h. After 15 min of fixation with 4 % paraformaldehyde, cells were permeabilized with 0.2 % Triton X-100 for 10 min at room temperature, then washed with phosphate-buffered saline (PBS), and blocked with 3 % bovine serum albumin (BSA). Coverslips were treated with primary antibodies at 4 °C overnight. Following 3 brief washes with PBS, the coverslips were incubated with appropriate secondary antibodies for 1 h. The samples were imaged by the Radiance 2100 Laser Scanning System (Bio-Rad, Hertfordshire, UK). DAPI was used to indicate the nuclei.

### Measurement of pHi

The pHi was measured using BCECF-AM in a Synergy H4 Hybrid Multi-Mode Microplate Reader (BioTek, Winooski, VT, USA) as previously described. Briefly, the cells were grown overnight in 96-well plates (Greiner Bio-One GmbH, Frickenhausen, Germany). After washing twice with serum-free DMEM, cells were incubated in serum-free DMEM containing 1 μM BCECF-AM for 30 min at 37 °C in 5 % humidified CO_2_. The cells were then washed with Ringer’s buffer (140 mM NaCl or Na gluconate, 5 mM glucose, 5 mM potassium gluconate, 1 mM calcium gluconate, 1 mM MgSO_4_, 2.5 mM NaH_2_PO_4_, 25 mM NaHCO_3_, 10 mM HEPES, pH 7.4) three times and incubated in Ringer’s buffer. BCECF-labeled cells were excited at 440 and 490 nm and emission was measured at 530 nm using a Multi-Mode Microplate Reader. Fluorescence excitation ratios were converted to pHi values using the high K^+^-nigericin method by linear regression to a calibration curve.

### Estimation of cell viability

SGC7901 cells were seeded in 96-well plates and cultured overnight, and then transfected with empty vectors or AE2 expression vectors. At different time points, 10 μl of MTT (5 mg/ml) was added to each well and incubated at 37 °C for 4 h. After carefully removing the supernatant from each well, an equal volume of DMSO (150 μl) was added to each well and mixed thoroughly. The absorbance from the plates was read at 490 nm with an ELISA reader.

### Transwell cell migration assay

The transwell cell migration assay was performed using transwell culture inserts (12-well, Corning, NY) according to the manufacturer’s instructions. SGC7901 cells were transfected with empty vectors or AE2 expression vectors for 48 h. The cells were seeded onto the upper chamber and allowed to migrate toward the lower face of the transwell culture inserts. Cells were then incubated at 37 °C for 20 h. The cells inside the upper chamber were scraped off. Migrated cells on the underside of the inserts were fixed in methanol, stained with gentian violet and counted for 5 random 100× fields per well. The cell number per image was determined by using Image J software.

### Immunohistochemistry

Paraffin-embedded gastric cancer samples and adjacent normal tissue samples were collected from surgical resection and endoscopic biopsy performed at Renji hospital and Ruijin hospital, Shanghai Jiao Tong University School of Medicine. None of the patients had undergone chemotherapy or radiotherapy before surgery. All the specimens were obtained according to protocols approved by the Committee on Clinical Investigations of the respective institutions. Tumor specimens were fixed in 4 % neutralized formaldehyde, embedded in paraffin and 4 μm sections were stained with hematoxylin and eosin (H&E). For immunohistochemistry, sections were subjected to antigen retrieval by incubation with citric acid (pH 6.0), and the endogenous peroxidase was blocked by treatment with 3 % H_2_O_2_ for 15 min. After overnight incubation with primary antibodies at 4 °C, tissue sections were incubated for 15 min at room temperature with an appropriate secondary antibody (MaxVision™ Kits) followed by 3,3′-diaminobenzidine staining, then counterstained with hematoxylin. Normal rabbit IgG isotypes served as negative control antibodies.

### Mouse tumor model and therapy

Four-week-old female athymic BALB/c nude mice were purchased from Shanghai Slac Laboratory Animal Co., Ltd. The principles governing the care and treatment of animals as stated in the guidelines for the Care and Use of Laboratory Animals, which was formulated by the Ministry of Science and Technology of the People’s Republic of China were followed. Mouse experiments were approved by the Animal Research Committee of Shanghai Jiao Tong University. Nude mice were subcutaneously injected into the flank with 5 × 10^6^ SGC7901 cells suspended in 0.1 mL 0.9 % NaCl. Twenty xenograft nude mice were prepared to investigate the effect of gastrin on gastric cancer growth. When tumors reached a volume of 200 mm^3^, mice were randomized into either the control group, which received 0.9 % NaCl subcutaneously, or into the therapeutic group, which received subcutaneous injections of gastrin (2 mg/kg, diluted in 0.9 % NaCl) twice daily. All mice were sacrificed after 20 days of subcutaneous injection. Tumor length and width were measured to calculate tumor volume (V) based on the following formula: V = length (mm) × width^2^ (mm)/2.

### Statistical analysis

SPSS 13.0 statistical package (SPSS, Inc., Chicago, IL, USA) was used to analyze the experimental data. The data were expressed as the mean ± SD. Comparisons between the two groups were performed with a *t*-test. The *χ*^2^ test was used to analyze the rank data. Multiple comparisons were performed with one-way analysis of variance (ANOVA). Kaplan-Meier survival curves of two groups were compared by log-rank test. Statistical differences were considered to be significant for *p* < 0.05.

## Results

### p16 interacted with both AE1 and AE2

**Fig. 1:**
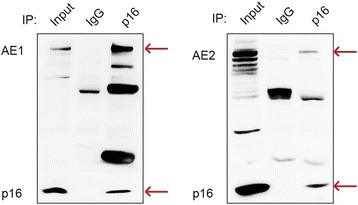
p16 interacted with both AE1 and AE2 proteins. An IP experiment was performed to examine endogenous interactions between AE1, AE2 and p16 in SGC7901 cells. Whole-cell lysates were immunoprecipitated with rabbit anti-p16 antibodies, followed by detection of AE1, AE2 and p16

Since AE1 has 80 % sequence similarity with AE2 in the C-terminal region [7, 23], and the C-terminus of AE1 can interact with p16 as we previously showed [17, 19], we hypothesized that p16 could also interact with the AE2 C-terminus. To test this possibility we performed an immunoprecipitation (IP) experiment using an anti-p16 antibody in SGC7901 cells. The results showed that the anti-p16 antibody precipitated detectable levels of both endogenous AE1 and AE2 (Fig. 1).

### AE1 and p16 interaction affected AE2 expression and function

**Fig. 2:**
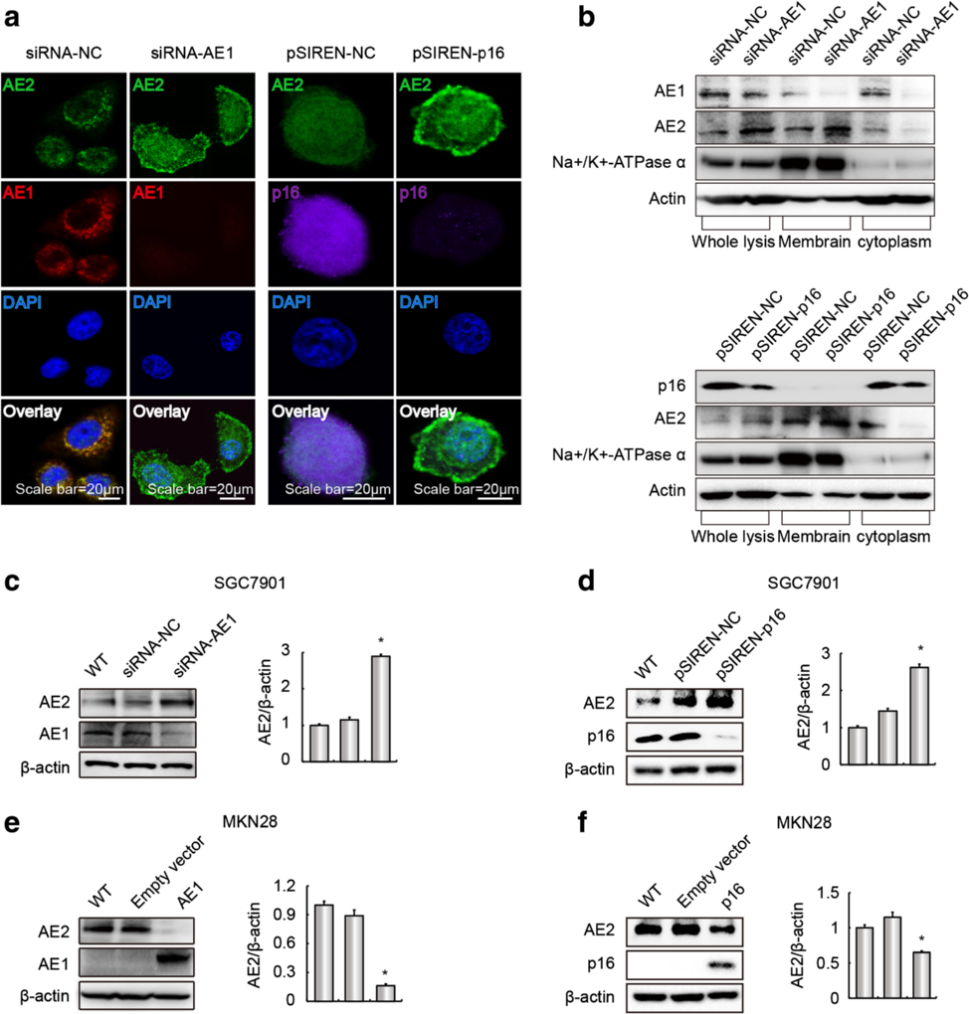
AE1 and p16 affected AE2 localization and expression in GC cells. **a** AE1 or p16 knockdown increased AE2 expression on the surface of SGC7901 cells. Nuclei were stained with DAPI (*blue*). **b** AE1 and AE2 expression in the membrane and cytoplasmic fractions of SGC7901 cells was detected by western blot. Cellular AE1 (*up panel*) and p16 (*bottom panel*) expression was suppressed by transfection with AE1- or p16-targeted siRNA or shRNA. **c**, **d** Silencing of AE1 (**c**) or p16 (**d**) increased AE2 expression in SGC7901 cells as verified by western blot. The ratio of AE2 protein abundance to that of β-actin was normalized to a value of 1.0 for SGC7901, *p < 0.05, compared with siRNA-NC/pSIREN-NC. **e**, **f** AE1 (**e**) or p16 (**f**) overexpression reduced AE2 abundance in MKN28 cells. The ratio of AE2 protein abundance to that of β-actin was normalized to a value of 1.0 for MKN28, *p < 0.05, compared with empty vectors. The values are expressed as mean ± SD of three different experiments performed in triplicate

We previously reported that AE1 and p16 were largely expressed in the cytoplasm of poorly-differentiated GC SGC7901 cells, but were absent in well-differentiated GC MKN28 cells [19, 20]. To further clarify the relationship among AE1, AE2 and p16, we down-regulated AE1 or p16 expression in SGC7901 cells by transfection with either AE1- or p16-targeted siRNA/shRNA plasmids. Confocal microscopy and cell fractionation experiments showed that individual knockdown of AE1 or p16 expression enhanced AE2 trafficking to the plasma membrane and nuclear distribution (Fig. 2a, b) and up-regulated AE2 expression (Fig. 2c, d). In contrast, overexpression of AE1 or p16 in MKN28 cells down-regulated AE2 expression (Fig. 2e, f).

### Cytoplasmic AE1/p16 promoted ubiquitin-dependent degradation of AE2 in GC cells

**Fig. 3:**
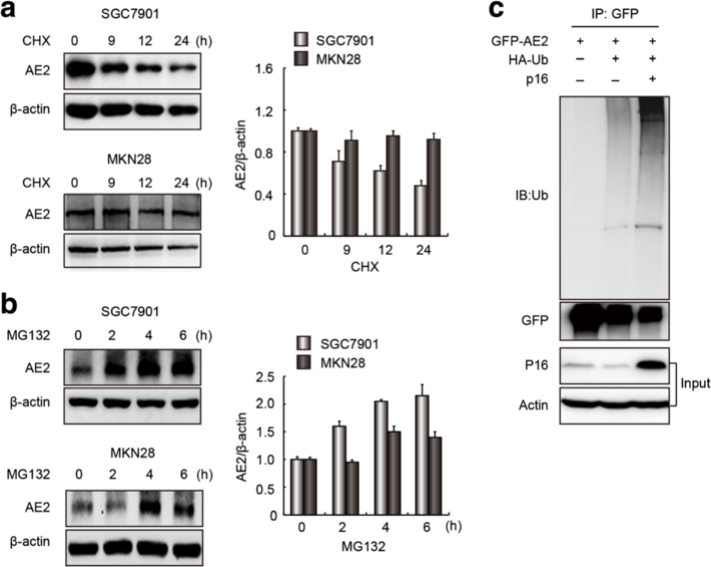
p16 enhanced ubiquitin-dependent degradation of AE2 protein. **a** AE2 expression in two GC cell lines treated with CHX (25 μg/ml for SGC7901 cells and 50 μg/ml for MKN28 cells) for the indicated times (*left*). The ratio of AE2 protein abundance to that of β-actin was normalized to a value of 1.0 for 0 h (*right*). Data are representative of experiments performed three times in triplicate. AE2 was more stable in MKN28 cells than in SGC7901 cells. **b** AE2 expression in two GC cell lines treated with 10 μM MG132 for the indicated times (*left*). Data are representative of experiments performed three times in triplicate. The ratio of AE2 protein abundance to that of β-actin was normalized to a value of 1.0 for 0 h (*right*). AE2 protein was more susceptible to degradation in SGC7901 cells than in MKN28 cells. **c** p16 enhanced ubiquitin-dependent degradation of the AE2 protein. HEK293T cells were co-transfected with vectors expressing pEGFP-AE2a, HA-Ub and p16, singly or in combination, as indicated. At 42 h after transfection, cells were treated with 10 μM MG132 for 6 h. Cell extracts were immunoprecipitated with anti-GFP antibodies to reveal polyubiquitination

We further speculated that the decreased levels of the AE2 protein could be due to the instability that is potentially associated with AE1 and p16 expression. To test this possibility, AE2 levels in SGC7901 and MKN28 cells were dynamically measured after the cells were treated with cycloheximide (CHX), an inhibitor of protein biosynthesis (25 μg/ml for SGC7901 cells and 50 μg/ml for MKN 28 cells), or the proteasome inhibitor MG132 (10 μM). Western blots showed that after blocking protein synthesis with CHX, the AE2 abundance in SGC7901 cells rapidly decreased compared to that in MKN28 cells (Fig. 3a). Moreover, AE2 protein levels were enriched more rapidly in SGC7901 cells than in MKN28 cells after treating the cells with MG132 (Fig. 3b). These results indicated that AE2 was unstable in poorly differentiated SGC7901 cells. To further test whether AE2 degradation was ubiquitin-dependent and was affected by p16 expression, a p16 expression vector was co-expressed with HA-ubiquitin (HA-Ub) in SGC7901 cells. IP experiments showed that polyubiquitin chains were present on GFP-AE2 (Ub-AE2) in cells that overexpressed p16 and were treated with MG132 (Fig. 3c). Taken together, these results indicated that p16 enhanced ubiquitin-dependent degradation of the AE2 protein and promoted AE2 instability in GC cells.

### AE2 suppressed GC growth by decreasing pHi

**Fig. 4:**
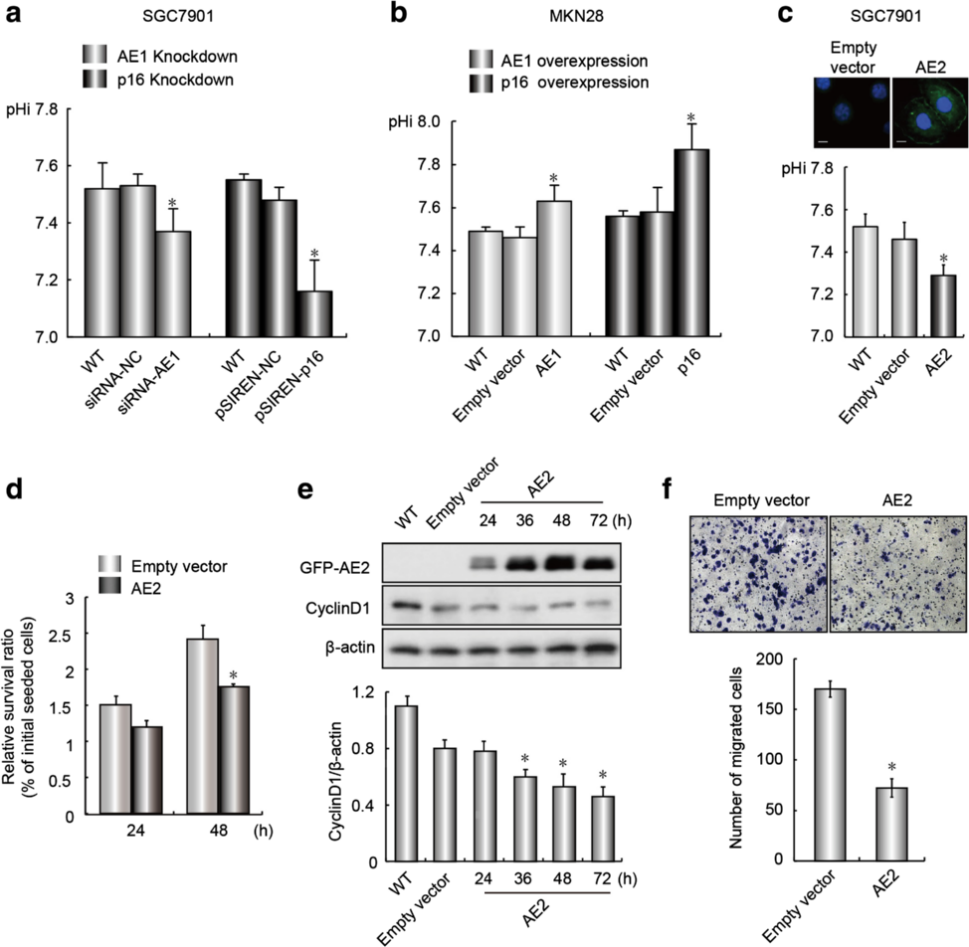
AE1 and p16 expression affected AE2 function. **a** Knockdown of AE1 or p16 expression reduced the pHi of SGC7901 cells. **b** AE1 or p16 overexpression increased the pHi of MKN28 cells. **c** AE2 overexpression reduced the pHi of SGC7901 cells. The pHi was measured using BCECF-AM. **d** AE2 overexpression decreased GC cell proliferation. **e** AE2 overexpression in SGC7901 cells decreased the abundance of cyclin D1 (*upper panel*) in a quantitative manner (*bottom panel*). **f** Transwell assay (*upper panel*) and statistical analysis (*bottom panel*). Values are expressed as mean ± SD of three different experiments performed in triplicate. *p < 0.05, compared with empty vectors or siRNA-NC/pSIREN-NC

Previous studies showed that the pHi was elevated in AE1- and p16-positive SGC7901 cells and that cellular alkalization favored cell proliferation [19, 24]. To explore whether the pHi was affected by AE1 or p16 expression, the pHi was measured after knockdown of AE1 or p16 expression in SGC7901 cells or over-expression of these two proteins in AE1- and p16-negative MKN28 cells. The results indicated that AE1 or p16 knockdown in SGC7901 cells resulted in a significant reduction in pHi compared with control cells (Fig. 4a). In contrast, AE1 or p16 overexpression in MKN28 cells significantly increased the pHi (Fig. 4b). These results suggested that AE2 might play a role in inhibition of GC cell proliferation. Forced AE2 expression in SGC7901 cells confirmed that the GFP-AE2 significantly decreased the pHi of the cells, and this decrease was accompanied by decreased cyclin D1 expression as well as GC growth and migration (Fig. 4c-f).

### Low levels of AE2 and high levels of AE1 and p16 correlated with poor prognosis of GC

**Fig. 5:**
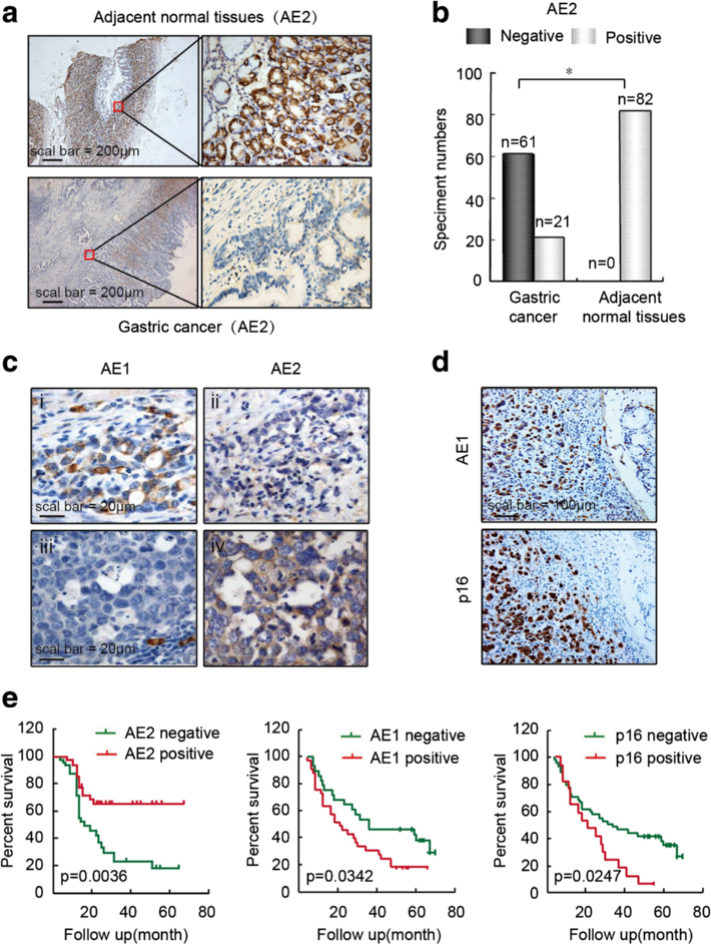
Low levels of AE2 and high levels of AE1 and p16 correlated with poor survival of GC patients. **a** Abundant amounts of AE2 were detected in adjacent normal gastric tissues (*upper panel*) from GC patients, which contrasted with the lack of AE2 expression in cancer tissues (*bottom panel*). **b** Statistical analysis of AE2 expression in GC tissues and adjacent normal tissues with n representing the number of specimens. **c** Expression of AE1 and AE2 in serial GC tissue sections. **d** AE1 and p16 expression in serial GC tissue sections. **e** Kaplan-Meier survival curves of overall survival of GC in terms of AE2, AE1 and p16 protein levels

To further explore the role of AE2 in inhibition of GC growth, AE2 expression in samples from 82 gastric cancer patients was detected by immunohistochemistry. AE2 was exclusively expressed in adjacent normal gastric tissues, but was significantly down-regulated in cancer tissues (Fig. 5a). Statistical analysis indicated that the expression frequency of AE2 in GC tissues was 25.6 % (21/82), which is significantly decreased compared with that in adjacent normal tissues (82/82) (Fig. 5b). Consistent with our molecular experiment results (Fig. 2), AE2 expression was negatively correlated with both AE1 and p16 expression in GC tissues (Fig. 5c, d). The clinicopathological analysis demonstrated that reduced AE2 expression was associated with poor differentiation of GC (Table 1). Most importantly, low levels of AE2 and high levels of AE1 and p16 were correlated with poor survival of GC patients (Fig. 5e).

**Table 1:** Relationship between AE2 expression and clinicopathological features of gastric cancer

Clinicopathological features	No.^a^	AE2 expression		*P* value
		Negative no. (%)	Positive no. (%)	
Mean ages (years)				
63.6 ± 12.8	174	119	55	
Age (years)				0.6247
≤ 65	87	61 (70.11)	26 (29.89)	
> 65	87	58 (66.67)	29 (33.33)	
Gender				0.4655
Male	107	71 (66.36)	36 (33.64)	
Female	67	48 (71.64)	19 (28.36)	
Differentiation				0.0005
Well or Moderately	72	39 (54.17)	33 (45.83)	
Poorly	101	80 (79.21)	21 (20.79)	
Lymph metastasis				0.7668
Negative	39	26 (66.67)	13 (33.33)	
Positive	133	92 (69.17)	41 (30.83)	
TNM staging				0.0179
I	20	9 (45.00)	11 (55.00)	
II–IV	130	93 (71.54)	37 (28.46)	
Lauren classification				0.0002
Intestinal type	66	37 (56.06)	29 (43.94)	
Diffuse type	82	69 (84.15)	13 (15.85)	

Values in parenthesis are percentage

^a^The data was partially analyzed due to the unparticular information

### Gastrin suppressed the growth of GC cells in vivo and in vitro through up-regulation of AE2 expression

**Fig. 6:**
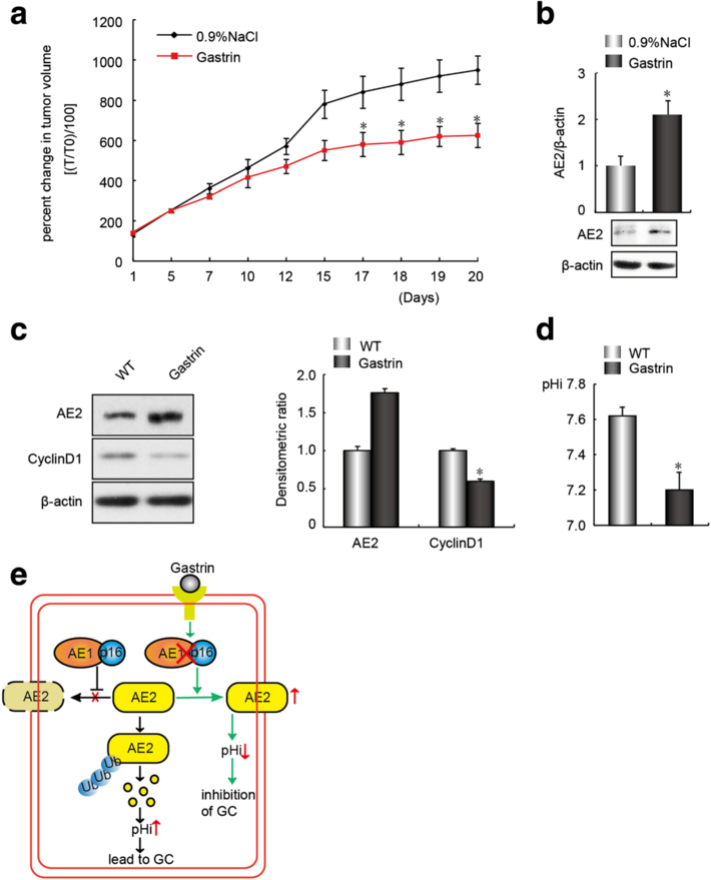
Gastrin suppressed GC growth in vivo and in vitro through AE2 up-regulation. **a** Gastrin induced inhibition of tumor cell proliferation. SGC7901 xenograft-bearing nude mice were treated with 0.9 % NaCl or gastrin. Tumor growth rates were determined by calculating the percentage of change in tumor volume (T) compared with initial tumor volume (T0). *p < 0.05, compared with control group (*n* = 10). **b** Gastrin increased AE2 abundance in tumor extracts. The ratio of AE2 protein abundance to that of β-actin was normalized to a value of 1.0 for the 0.9 % NaCl group. *p < 0.05, compared with 0.9 % NaCl group (*n* = 10). A representative immunoblot is presented below. **c** Gastrin induced up-regulation of AE2 protein expression and down-regulation of cyclin D1 protein expression. The ratio of AE2 and cyclin D1 protein abundance to that of β-actin was normalized to a value of 1.0 for the SGC7901 group (*n* = 3), *p < 0.05, compared with the SGC7901 group. **d** Cellular acidification occurred in SGC7901 cells after incubation with 10^−7^ M gastrin for 24 h. Data are representative of experiments performed three times in triplicate, *p < 0.05, compared with untreated SGC7901 cells. **e** Model for gastrin-induced inhibition of GC through up-regulation of AE2 levels, which were decreased by AE1/p16. In GC cells, intracellular retention of AE1 and cytoplasmic sequestration of p16 lead to increased AE2 ubiquitin-dependent degradation, as well as reduced total and plasmalemmal abundance of AE2. The resulting reduction in AE2-mediated anion exchange activity may result in cellular alkalinization, leading to increased cyclin D1 expression. Gastrin up-regulates AE2 expression by blocking formation of the AE1/p16 complex. Enhanced AE2-mediated Cl^−^/HCO_3_^−^ exchange activity acidifies GC cells that in turn retards cell growth

We previously reported that gastrin inhibited the growth of AE1- and p16-positive GC cells [25]. To investigate a potential role for AE2 in mediating gastrin-induced GC suppression, a SGC7901 xenograft mouse model was established by subcutaneous injection of 5 × 10^6^ SGC7901 cells. After the initial tumors reached 200 mm^3^, animals were randomized into control and experimental groups, with the control group receiving 0.9 % NaCl, and the experimental group receiving subcutaneous injections of gastrin (2 mg/kg, diluted in 0.9 % NaCl) twice daily for 20 days. Tumor suppression was observed after treatment with gastrin for 17 days (Fig. 6a), and was accompanied by increased AE2 protein levels in tumor extracts on day 20 (Fig. 6b). To confirm the role of gastrin in AE2 up-regulation, AE2 expression was detected by western blot after cells were treated with 10^−7^ M gastrin for 3 days. The results showed that gastrin up-regulated AE2 expression, which was accompanied by down-regulation of cyclin D1 expression (Fig. 6c) and a reduction in pHi (Fig. 6d).

## Discussion

The sodium-independent Cl^−^/HCO_3_^−^ transporters that make up the AE family, together with other ion carriers, are involved in pHi regulation [10, 26, 27]. Under physiological conditions, AE family proteins are generally activated by intracellular alkalosis. All AE members share three common structural domains: an N-terminal cytoplasmic domain, a transmembrane domain and a C-terminal cytoplasmic domain, yet their molecular weights differ (AE1: ~95 kDa and AE2: ~170 kDa) [7]. The AE1 gene encodes full-length erythroid AE1 and a shorter kidney AE1 that in humans initiates at Met66 [28]. Intracellular trafficking of AE1 is mediated via interactions with other proteins. For instance, interplay between AE1 and p16 facilitates AE1 trafficking to the plasma membrane, and also promotes p16 nuclear transport in AE2-positive HEK293T or K562 cells. Moreover, AE1 expression induces differentiation of the myelogenous leukemia cell line K562 [19, 24]. These results suggest that in several cell types, such as renal tubular epithelial cells or hematopoietic cells, AE1 can be transported to the plasma membrane, and that AE1 and p16 play a mutually cooperative role in HEK293T and K562 cells. AE2 and p16 proteins were also normally located at the plasma membrane and in the nucleus, respectively, in these cells [17, 24]. On the other hand, AE1, AE2 and p16 mRNAs could be detected under physiological conditions in gastric epithelial cells. The AE2 mRNA was translated into proteins, while translation of both AE1 and p16 was normally silenced by several factors, including miR-24 and gastrin [24, 25]. In contrast, under pathologic conditions such as achlorhydria and *H. pylori* infection, AE1 expression is significantly induced and causes a large accumulation of AE1 in the cytoplasm of gastric epithelial cells [19, 22]. Such cells lack a route for AE1 membrane trafficking, leading to sequestration of AE2 in the cytoplasm that arises in response to interactions between cytoplasmic AE1 and p16. The cytoplasmic AE2 protein may be misfolded and thus more sensitive to ubiquitin-dependent degradation [29, 30]. These results suggested that AE1 and AE2 do not normally coexist in the cytoplasm of cells, although AE2 overexpression can produce protein expression rates that exceed those of degradation. This undegraded AE2 protein could coexist with AE1 in the cellular cytoplasm.

AE2 is known to be widely expressed in most cell types, and has a particularly high expression level in gastric parietal cells. Nevertheless, how AE2 protein is removed from cells is unclear. Here we demonstrated that AE2 is degraded by the ubiquitin proteasome pathway in GC cells. Aberrant AE2 expression levels could cause total or partial dysfunction in regulating pHi [7]. Several studies demonstrated that cytoplasmic pH plays crucial roles in controlling DNA synthesis, cell growth, proliferation, differentiation, oncogenesis and malignant transformation [31]. In addition, there is increasing evidence to support that cancer cells have ‘malignant’ alkaline pHi, which is consistent with our previous findings [32]. Thus, intracellular alkalinization may be an important hallmark of GC tumor cells [33, 34]. We speculated that in GC, impaired AE2 expression in turn elevates the pHi and reduces acid secretions via interactions with AE1 and p16, which could worsen achlorhydria syndromes and promote GC progression (Fig. 6e). AE1 was previously demonstrated to be an unexpected factor that is responsible for p16 cytoplasmic sequestration and is associated with both tumor progression and poor prognosis [17, 18]. As such, the nuclear distribution of AE2 requires further investigation.

## Conclusions

Here we demonstrated that ectopic expression of AE2 together with AE1 and p16 expression is an important pathogenic factor in the development of GC, and that dysfunctional AE2 can be degraded through a ubiquitin-dependent pathway. Gastrin affects AE2 expression and thus could be a potential candidate drug for targeting therapy for AE1- and p16-positive GC. Our findings will bring new perspectives on future clinical treatments for GC.

## Abbreviations

AE1: Anion exchanger 1
AE2: Anion exchanger 2
BCECF-AM: 2′,7′-bis-(2-carboxyethyl)-5-(and-6)-carboxyfluorescein acetoxymethyl ester
CHX: Cycloheximide
GC: Gastric cancer
IP: Immunoprecipitation
MTT: 3-(4,5-dimethyl-2-thiazolyl)-2,5-diphenyl-2-H-tetrazolium bromide
pHi: Intracellular pH
SLC4A1: Solute carrier family 4, anion exchanger, member 1
SLC4A2: Solute carrier family 4, anion exchanger, member 2
